# The role of gastric cancer patient-derived organoids in precision medicine: Clinicians’ perspective

**DOI:** 10.1016/j.stemcr.2026.103006

**Published:** 2026-07-09

**Authors:** Tharindie N. Silva, Harleen Kaur, Tharani Krishnan, David S. Liu, Margaret Lee, Markus Trochsler, Nicholas J. Clemons, Josephine A. Wright, Susan L. Woods, Gastric Cancer Organoid Survey group, David S. Liu, Ignace De Hingh, Neda Nikolic, Megan Barnet, Manjunath Siddaiah-Subramanya, David I. Watson, Marcello Guaglio, Diane Goéré, Chirag Patel, Conrad Stranz, Markus Trochsler, Philip Game, Tim Bright, Lauren Kennedy, Li L. Kuan, Guy Maddern, Shalvin Prasad, Matthew Watson, Cameron Schauer, Harleen Kaur, Michael W. Hii, Marina Puchinskaya, Radu Vidra, William Butterworth, Karez Namiq, Jane M. Andrews, Tudor Boloş, Emiliana-Viorica Larionesi, Matthew A.R. Stokes, Daniel Worthley, Jessie A. Elliott, Andreas R.R. Weiss, Yit J. Leang, Benjamin Tan, Munir Tarazi, Hien Le, Stephen Kunz, Di Maggio Francesco, Charles J. Rayner, Martin Harutyunyan, Patricia Kaazan, Richard Heddle, Nagendra N. Dudi-Venkata, Vijay Abraham, Julian Süsstrunk, Aniket Nadkarni, Panagiotis J. Vlachostergiostergios, Shantanu Joglekar, Oktay Irkorucu, Brian Stein, Semra D. Atici, Vladimir Andelkovic, Mariella Spalato-Ceruso, Sharif Ahmed, Ryan Newbold, Michele Ghidini, Motohiro Imano, Birute Brasiuniene, İmdat Eroğlu, Fabio M. Oddi, Christopher M. Jones, Martynas Luksta, Samuel Smith, Sharon Pattison, Shailesh V. Shrikhande, Tharani Krishnan, Savio G. Barreto, Manju D. Chandrasegaram, Bartłomiej Tomasik, Andrew L. Nathanson, Piotr T. Wysocki, Sam Banks

**Affiliations:** 1 Adelaide Medical School, Adelaide University, Adelaide, SA, Australia; 2 Precision Cancer Medicine Theme, South Australia Health and Medical Research Institute, Adelaide, SA, Australia; 3 Department of Surgery, Adelaide University, The Queen Elizabeth Hospital Adelaide, Woodville South, SA, Australia; 4 College of Medicine and Public Health, Flinders University, Bedford Park, SA, Australia; 5 Department of Medical Oncology, Flinders Medical Centre, Bedford Park, SA, Australia; 6 Division of Cancer Surgery, The Peter MacCallum Cancer Centre, Melbourne, VIC, Australia; 7 Victorian Interventional Research and Trials Unit, Department of Surgery, The University of Melbourne, Heidelberg, VIC, Australia; 8 Upper Gastrointestinal Surgery Unit, Austin Health, Heidelberg, VIC, Australia; 9 Department of Medical Oncology, Western Health, Melbourne, VIC, Australia; 10 Cancer Research Division, Peter MacCallum Cancer Centre, Melbourne, VIC, Australia; 11 Sir Peter MacCallum Department of Oncology, The University of Melbourne, Melbourne, VIC, Australia

**Keywords:** gastric cancer, organoids, precision medicine, clinician perspective survey

## Abstract

Organoid technology holds great potential and hope for personalized treatment in gastric cancer (GC). To date, clinicians’ views on this technology for precision oncology have not been reported despite these insights being invaluable to understanding how organoids may be effectively integrated into clinical practice. Hence, we explored clinicians’ knowledge and perception of organoid research and its potential clinical utility in GC treatment. Clinicians believed that organoids would be most useful for guiding adjuvant treatment in patients who fail neoadjuvant therapy and emphasized the importance of obtaining results within 4–6 weeks of surgical resection. Clinicians also expressed concerns and suggestions related to current research methodologies and highlighted the ethical and political aspects of organoid-guided personalized medicine. Overall, most clinicians perceived the value in the use of organoids to guide personalized treatment in GC and provided important insights into how this approach could be best implemented in clinical practice in the future.

## Highlights

• Clinicians reported positive attitudes toward organoid-guided personalized treatment
• Guiding adjuvant therapy in patients who fail neoadjuvant treatment was highlighted
• Organoid drug assay results within 4–6 weeks indicated as optimal
• Clinical trial validation of organoid drug assay a key priority for the field

## Teaser

In this article, Silva and colleagues report that clinicians treating gastric cancer recognize the promise of organoid technology for personalized treatment in the future, particularly following failed standard care neoadjuvant therapy. They highlighted methodological, ethical, and political challenges that must be addressed to integrate this approach into future clinical practice.

## Introduction

Derived from adult stem cells, organoids are an *in vitro* 3D cell culture system that mimic native tissue architecture and function (Sato et al., 2009). This is a significant improvement from traditional 2D cell culture models that have vastly contributed to the biomedical field, as organoids offer a more physiologically relevant model (Silva et al., 2024). Organoids have been successfully established from many cancers, including breast, lung, colorectal, and pancreatic cancers (Below et al., 2022; Bhatia et al., 2022; Dekkers et al., 2020; Ganesh et al., 2019; Kim et al., 2019; Narasimhan et al., 2020; Sachs et al., 2019; Tiriac et al., 2018; Vlachogiannis et al., 2018).

Gastric cancer (GC) is a highly aggressive disease that is frequently diagnosed in advanced stages. Despite recent advances in targeted agents and immunotherapy for this cancer, patients often progress, and the overall prognosis is poor (Takei et al., 2022). Novel therapeutic approaches to better personalize treatment for these patients are needed. Over the past 5–10 years, GC patient-derived organoid biobanks that recapitulate the heterogeneity of gastric adenocarcinomas have been established internationally (Chen et al., 2024; Gao et al., 2018; Li et al., 2019; McDonald et al., 2023; Schmäche et al., 2024; Seidlitz et al., 2019; Steele et al., 2019; Vlachogiannis et al., 2018; Xu et al., 2024; Yan et al., 2018; Yoon et al., 2023; Zhang et al., 2023; Zhao et al., 2024; Zu et al., 2023). *In vitro* treatment of GC patient-derived organoids has been retrospectively shown to predict clinical patient-specific responses to the same chemotherapy agents and targeted therapeutics, although study sizes are relatively small (Gao et al., 2018; Schmäche et al., 2024; Steele et al., 2019; Vlachogiannis et al., 2018; Yan et al., 2018; Yoon et al., 2023; Zhao et al., 2024; Zu et al., 2023). For example, organoids from pre-treatment biopsies of patients with gastroesophageal cancer (*n* = 13 exploratory, *n* = 13 validation cohort) were established to study responses to neoadjuvant FLOT (5-fluorouracil, leucovorin, oxaliplatin, docetaxel) chemotherapy and correlated with clinical outcomes (Schmäche et al., 2024). The study demonstrated that organoid testing accurately predicted clinical response in the validation cohort, with high sensitivity (90%), specificity (100%), and accuracy (92%). This is consistent with larger cohorts in colorectal cancer that demonstrated the predictive power of drug responses from patient-derived organoids for progression-free survival, but studies have not always been positive for oxaliplatin-containing regimens in retrospective comparisons (Wang et al., 2023). Furthermore, there are numerous clinical trials registered to test the effectiveness of patient-derived organoids in predicting treatment responses in GC, e.g., NCT05652348, NCT05351398, NCT05842187, which aim to test the clinical utility of GC organoids for precision oncology. If successful, these approaches could potentially provide clinicians with a tool for personalized treatment decision-making, decreasing unnecessary side effects and improving survival outcomes for patients with GC.

The public and patient perception of organoid research has been explored in recent studies, which have demonstrated a generally supportive attitude toward this technology, subject to responsible governance, ethics, commercialization, healthcare access, and provision of informed consent (Boers et al., 2018; Bollinger et al., 2021; Haselager et al., 2020; Ravn et al., 2023). However, we lack studies exploring the clinicians’ perspective on organoid-guided treatment in patients with cancer. With the expansion of organoid technology toward precision medicine clinical trials, this will be key to its effective integration into the clinical setting to provide meaningful benefits for patients with cancer. We conducted a survey of clinicians involved in the care of patients with GC to better understand their knowledge and perception of patient-derived organoid research, as well as their views on appropriate patient selection, required turnaround times for organoid testing, and anticipated challenges of using patient-derived organoids to guide GC treatment. These insights into how organoid technology might be applied in the real-world clinical setting will help shape future research and clinical trial design to increase the likelihood that organoids may be used to improve patient outcomes in the future.

## Results

Here, we report on four themes—the knowledge of clinicians on organoid technology, preferred patient selection for organoid use, time sensitivity and need for results in real-world patient care, and the potential and feasibility of organoids in future clinical practice.

### Demographics

A total of 87 complete responses were received from clinicians across 23 countries spanning four continents: Oceania, 51; Europe, 26; Asia, 9; and North America, 1 (Table 1; Figure S1). The majority were surgeons (*n* = 44, 50.6%), followed by medical oncologists (*n* = 20, 23%), gastroenterologists (*n* = 7, 8%), surgical trainees (*n* = 5, 5.7%), radiation oncologists (*n* = 2, 2.3%), and clinical oncologists (*n* = 2, 2.3%), while the remaining professions (medical oncologist trainee, palliative medicine specialist and rural generalist, medical officer, radiologist, junior doctor, intensive care specialist, and surgical oncologist) each accounted for one response.

**Table 1:** Demographics of survey respondents

A total of 87 survey respondents	

**Country, *n* (%)**	

Australia	49 (56.3)
United Kingdom	7 (8)
Italy	3 (3.4)
Turkey	3 (3.4)
New Zealand	2 (2.3)
France	2 (2.3)
Germany	2 (2.3)
Lithuania	2 (2.3)
Poland	2 (2.3)
Romania	2 (2.3)
Other	13 (14.9)

**Profession, *n* (%)**	

Surgeons	44 (50.6)
Medical oncologists	20 (23)
Gastroenterologists	7 (8)
Surgical trainees	5 (5.7)
Radiation oncologists	2 (2.3)
Clinical oncologists	2 (2.3)
Other	7 (8)

### Clinicians’ knowledge of organoid technology

**Figure 1:**
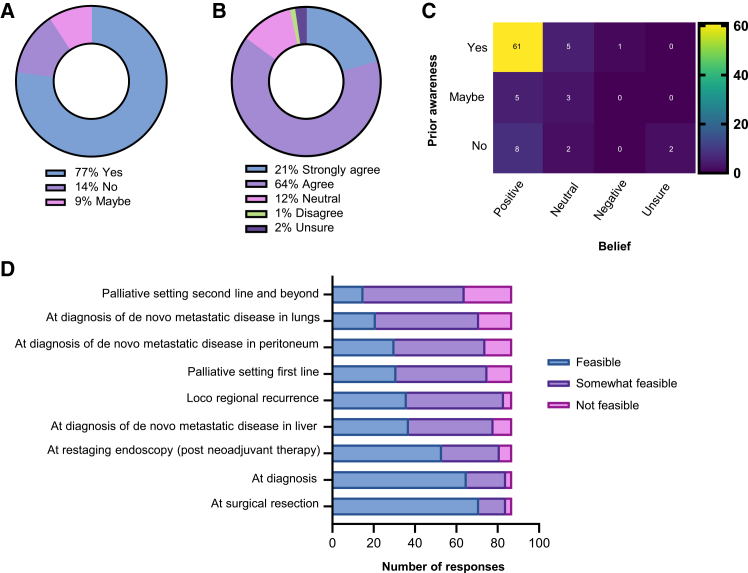
Current use of organoids in clinical practice and feasibility of biopsy acquisition (A) Awareness of research that demonstrates the use of organoids in predicting treatment response for cancer patients. (B) Distribution of clinicians’ agreement levels regarding the use of organoids in predicting patients’ responses to specific drugs. (C) Heatmap showing clinicians’ perceptions of the usefulness by prior awareness of organoid research in predicting treatment response for cancer patients. Rows represent awareness categories (“yes,” maybe,” and “no”), and columns represent responses to the usefulness question (“strongly agree,” “agree,” “neutral,” “unsure,” “disagree,” and “strongly disagree”). Color intensity reflects the number of respondents in each cell. (D) Feasibility of taking additional biopsies for organoid culture (research purposes) at nine different clinical time points.

A lay term description was presented at the start of the survey: “Organoid culture is a method used to grow mini organs in a dish, derived from stem cells. Tumor organoids are derived from cancer cells and represent personalized models that can be utilized for research and drug testing.” Out of the 87 clinicians, 77% (*n* = 67) were aware of research that demonstrates the use of organoids in predicting treatment response for cancer patients, while 9% (*n* = 8) answered “maybe,” reflecting uncertain knowledge, and 14% (*n* = 12) were not aware of any organoid research for treatment prediction (Figure 1A).

The majority of clinicians believed organoids could predict how a patient’s cancer would respond to specific drugs, where 21% (*n* = 18) strongly agreed, 64% (*n* = 56) agreed, 12% (*n* = 10) recorded neutral, 1% (*n* = 1) disagreed, and 2% (*n* = 2) were unsure (Figure 1B). When matched with their prior awareness of organoid research, of those who were aware of organoid research, 91% had positive beliefs (strongly agreed or agreed), with 8% being neutral, and 2% in disagreement, while among those clinicians who were uncertain or unaware of organoid research, 65% (*n* = 13) were optimistic, with 35% (*n* = 7) being unsure (chi-square test, χ^2^ = 19.97, df = 6, *p* = 0.0028) (Figure 1C).

### Patient selection for organoid use

**Figure 2:**
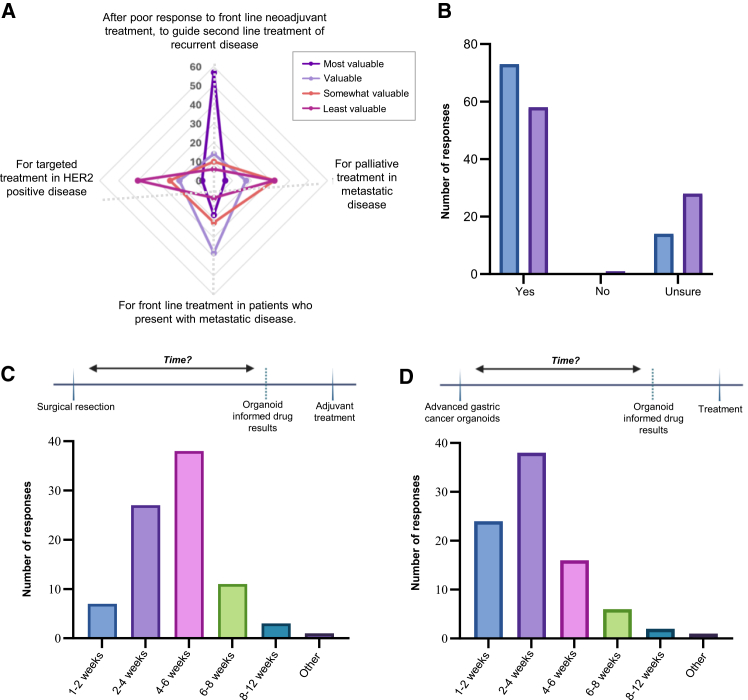
Patient selection and time sensitivity for organoid drug assay results in clinical practice (A) Radar plot illustrating clinicians’ ratings of the perceived value of organoid-guided treatment in four clinical scenarios. Plot axes represent the clinical scenarios, while radial distance reflects the number of clinicians selecting each value category. (B) Bar graph showing the number of clinicians who would consider using organoids to guide treatment choice across two clinical settings. The first bar (blue) is for patients who fail first-line standard care treatment, and second bar (purple) is for patients in the refractory palliative setting after failing standard-of-care treatments. (C) Bar chart depicting how quickly clinicians would like to receive organoid-informed drug results after surgery to guide adjuvant treatment. (D) Bar chart depicting how quickly clinicians would like to receive organoid-informed drug results to guide treatment in patients with advanced gastric cancer.

The next four questions were focused on identifying at which point in clinical care of a GC patient, the organoid-guided personalized treatment was viewed by clinicians as most feasible and useful. 82% (*n* = 71) reported that it is most feasible (“feasible” and “somewhat feasible”) at surgical resection, closely followed by time of diagnosis (75%, *n* = 65) and at restaging endoscopy (post-neoadjuvant therapy) (61%, *n* = 53). The remaining clinical time points were predominantly rated as somewhat feasible (Figure 1D). Respondents were given four clinical scenarios to rank based on where organoid models could provide the most value in guiding treatment options. “To guide adjuvant treatment after poor response to neoadjuvant therapy to prevent recurrent disease” was ranked as the most valuable scenario (66%, *n* = 57), followed by “for first-line treatment in patients who present with metastatic disease” (44%, *n* = 38), “for palliative treatment in metastatic disease” (37%, *n* = 32), and “for targeted treatment in human epidermal growth factor receptor 2 (*HER2*)-positive disease” (46%, *n* = 40), which was ranked least valuable (Friedman χ^2^(3) = 80.34, *p* < 0.0001) (Figure 2A).

Consistent with the scenario ranking, 84% (*n* = 73) of clinicians reported they would consider using organoid drug testing to guide treatment choice for patients who fail first-line standard-of-care treatment (Figure 2B). While the ranking question looked at clinical scenarios and the follow-up asked if the clinicians would likely implement organoid-guided treatment, the same patient group was consistently preferred. 67% of clinicians would consider organoid drug testing to guide treatment decisions for patients in the refractory palliative setting after failing standard-of-care treatments, where 33% were “unsure” (Figure 2B). This shows that clinicians indicated they would be more likely to use organoid-guided drug testing for palliative care patients with poor response to first-line treatment compared to the previous ranking question where first-line palliative treatment in metastatic disease was only viewed as the most valuable use of organoid technology for a very small population of clinicians (7%). Overall, clinicians favored the use of organoid technology to guide treatment at second line for patients with poor response to the first-line treatment, compared with the refractive setting (chi-square test, χ^2^ = 7.38, df = 2, *p* = 0.025).

### Time window to report organoid drug response

The time period between diagnosis, treatment initiation, and detection of response to standard care or disease recurrence differs for different cancers, disease stages, subtypes, and each patient. To understand the likely time frame for clinically actionable organoid-guided treatments for GC, clinicians were asked two questions related to the time sensitivity of results, with a few predefined time frames given as options. These options were based on previous studies that looked at the optimal timing for initiating adjuvant or neoadjuvant chemotherapy for GC patients (Huang et al., 2019; Maeng et al., 2024; van Erp et al., 2020) or the turnaround times reported in current organoid studies for generating drug assay results (Schmäche et al., 2024; Zhao et al., 2024). If organoid drug results guide treatment decisions in the adjuvant setting after surgical resection, 44% (*n* = 38) of clinicians would consider 4–6 weeks as acceptable for receiving organoid-informed drug results vs. 31% (*n* = 27) 2–4 weeks, and 12.6% (*n* = 11) 6–8 weeks (goodness-of-fit chi-square, χ^2^(5) = 82.5, *p* < 0.0001) (Figure 2C). The maximum time from diagnosis to the availability of organoid-informed drug testing results deemed useful to guide the choice of treatment for patients with advanced GC was shorter at 2–4 weeks (*n* = 38, 43.7%), followed by 1–2 weeks (*n* = 24, 27.6%) and 4–6 weeks (*n* = 16, 18.4%), with other time frames receiving fewer than 10 responses (goodness-of-fit chi-square, χ^2^(5) = 74.5, *p* < 0.0001) (Figure 2D).

### Clinical considerations for choice of drug for organoid-guided treatment

**Figure 3:**
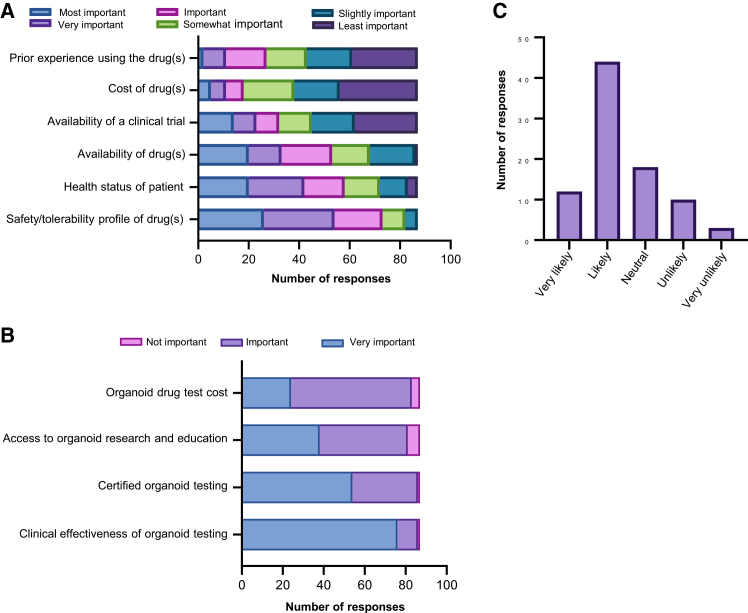
Potential and feasibility of organoids in clinical practice (A) Distribution of clinician responses ranking the relative importance of factors influencing organoid-guided treatment selection. (B and C) Importance of factors influencing the incorporation of organoid test results into routine treatment decision-making; response items are truncated for brevity (B). Distribution of clinician responses regarding the likelihood of organoid drug testing being incorporated into standard care gastric cancer personalizing treatment within the next decade (C).

The majority, 62% (*n* = 54) of clinicians, ranked the safety and tolerability profile of drug(s) as the most or very important when considering choice of chemotherapeutics for use in GC patients based on organoid drug assays (Friedman test, χ^2^(5) = 102.4, *p* < 0.0001) (Figure 3A). Health status of the patient and availability of drug(s) were also highly valued, with many clinicians selecting them as most (both, 23%, [*n* = 20]) or very important (25% [*n* = 20] and 15% [*n* = 13], respectively] (Figure 3A). In contrast, the cost of drug(s) was generally seen as least important, with the largest number of clinicians (36%, *n* = 31) ranking it in this category. Prior experience using the drug(s) and availability of a clinical trial were of moderate importance. The participants ranked these factors differently in importance (Friedman test, χ^2^(5) = 102.4, *p* < 0.0001). Overall, these findings suggest that clinicians place the greatest emphasis on safety profile of the drug, health status of the patient, drug availability, and clinical trial evidence, rather than on cost or prior experience when making treatment decisions.

### Potential and feasibility of organoids in future clinical practice

The final section of the survey was focused on factors required for clinicians to be confident in using organoid drug assay data in clinical practice for GC treatment decision-making in the future. “Demonstrated clinical effectiveness of organoid test” stood out as a very important factor to 87% (*n* = 76) of respondents (chi-square test, χ^2^(6) = 72.44, *p* < 0.0001) (Figure 3B). Ratings of “increased access to organoid research data and education” and “clinical laboratories certified organoid testing processes” were more mixed, although most considered these factors “important” (49% and 37%, respectively) or “very important” (44% and 62%, respectively). In contrast, “organoid drug test cost” was more often considered “important” (68%), rather than “very important” (28%). Overall, very few respondents rated any factor as not important, suggesting that all factors were generally viewed as relevant. Over half (51%, *n* = 44) of the clinicians believed it was likely that organoids would become a standard care tool within the next 10 years, with 14% (*n* = 12) rating it as “very likely.” Only 15% (*n* = 13) rated it as “unlikely” or “very unlikely,” suggesting that most clinicians are confident about organoid technology being adapted into future clinical care (Figure 3C).

### Qualitative survey comments

In addition to the structured survey questions, clinicians were allowed to provide additional comments at three different sections of the survey. These questions aimed to explore additional factors that clinicians believe should be considered when using organoid drug tests in standard GC treatment. Of 87 clinicians, 24 provided additional comments. These included surgeons (*n* = 14), surgical trainees (*n* = 2), medical oncologists (*n* = 4), radiation oncologists (*n* = 1), gastroenterologists (*n* = 2), and clinical oncologists (*n* = 1). From 42 written answers, six key themes emerged: research, clinical, cost, access, policy, and ethical, as illustrated in the sunburst chart (Figure S2).

#### Theme 1: Research

Eight sub-themes were identified, reflecting research considerations that clinicians believe must be addressed to enable the integration of organoid drug testing into clinical practice. This included standardization of organoid drug testing practices, emphasizing known problems with variability in current organoid drug assays. The time required to complete organoid drug testing, lab to patient proximity, and culture success rates, particularly those post-treatment, were also highlighted. While researchers are working to establish organoid co-culture models with the addition of other cell populations (immune, stromal, etc.), clinicians highlighted this lack of an immune system in current organoid studies as a limitation that prevents personalized immunotherapy treatments. Another suggestion was to examine the utility of organoids to predict patient treatment responses at various stages of disease (primary resectable, metastatic, second line, and beyond).

#### Theme 2: Clinical

Many clinicians emphasized the need for a clinical trial evaluating organoid-guided GC treatment to assess integration into standard care, while one clinician suggested that this trial should demonstrate superior patient outcomes, beyond the correlation between organoid drug tests and corresponding patient clinical response to the same drug. Another comment highlighted the importance of providing proceduralists, such as endoscopists, with information on organoid test availability and testing procedures at the point of diagnosis. Among these suggestions, clinicians also noted that biopsies from distant organ metastases are more difficult to obtain due to increased biopsy risk, hence limiting personalized treatment for some patients.

#### Themes 3 and 4: Access and cost

Access to organoid drug testing was another major concern among clinicians, with questions raised about how organoid drug testing could be accessed by patients in metropolitan and regional areas, as well as eligibility criteria determining who could access such a service. Clinicians were also interested in the cost of the organoid drug testing process, with many agreeing that cost is a main factor that would influence its integration into standard practice, and one added that it’s “too expensive and slow, feeling that instead drugs are getting much better, much faster.” While organoids can be used to assess novel drugs, the aim of organoid drug testing in this context is to personalize treatment for patients with cancer, utilizing current standard-of-care drugs, rather than finding new drugs. Clinicians also expressed their interest in cost transparency, another commenting that the “availability/access to potential drugs, especially in New Zealand, where they may not always be available without personal cost to patient” was another factor that may influence the incorporation of organoid-guided drug test into GC treatment planning.

#### Themes 5 and 6: Policy and ethics

For organoid drug testing to be successfully introduced into standard care treatment, clinicians highlighted the need to consider not only methodological and clinical validity but also political/policy-related and ethical factors, for example, assessing medico-legal validity for organoid drug testing integration into standard practice. One clinician noted that “Australia is far behind in research and implementation of research due to political ignorance,” suggesting that political support for organoid drug testing may smooth transfer of this technology to clinical care. These factors will remain critical even with positive clinical trial evidence.

One comment summed up all six themes discussed above, “Organoids hold strong potential, but wider adoption will depend on faster turnaround times, standardized drug-response reporting, and clear evidence from prospective clinical studies. Integration with molecular profiling may further improve their predictive value,” with another adding, “In an ideal world, organoids should be grown from every patient as a routine part of workup.” Overall, these comments provide important guidance for researchers conducting organoid drug testing studies and/or clinical trials, emphasizing the need to address these additional factors for successful clinical implementation.

## Discussion

The growing need for personalized treatment approaches for patients with GC has highlighted the potential for including organoid drug testing to guide therapeutic choice. Although researchers are advancing the organoid field, understanding clinicians’ perspectives of the technology is critical, as they are at the forefront of patient care. Hence, this study provides insights from a diverse group of 87 GC clinicians from 23 countries on patient-derived organoids and their potential clinical use to personalize GC treatment. To the best of our knowledge, this is the first study to provide clinicians’ perspectives on organoid research.

We found that 77% of clinicians were aware of organoid-related research, with more than 80% perceiving organoids as a valuable tool to predict a patient’s response to different cancer drugs, showing positive attitudes toward organoid-guided personalized treatment.

The treatment of patients with GC depends on various factors such as cancer stage, suitability for surgical resection and individual patient co-morbidities, and performance status (Bonelli et al., 2019). There are various potential clinical time points at which organoids could be utilized to personalize treatment, which has implications for organoid platform development to guide treatment decisions. Current studies have acquired tumor tissue from surgical resection (Seidlitz et al., 2019; Steele et al., 2019; Yan et al., 2018), at surveillance endoscopy (McDonald et al., 2023; Schmäche et al., 2024; Vlachogiannis et al., 2018; Yoon et al., 2023) or from ascitic fluid drainage (Li et al., 2019) to then correlate organoid drug responses with either first-line or second-line chemotherapy treatment responses. For example, recent studies included matched patient and organoid drug responses (25 patients), with tissue samples collected from treatment-naive patients to assess neoadjuvant treatment (Schmäche et al., 2024; Zhao et al., 2024). The questionnaire indicated that clinicians viewed patients with poor response to first-line neoadjuvant therapy as the population most likely to benefit from organoid-guided approaches, with 84% indicating they would consider this strategy to inform personalized adjuvant treatment, particularly given recent findings demonstrating that a poor response to neoadjuvant treatment predicts minimal benefit to the same regimen in the adjuvant setting (Investigators, 2025). Further strengthening this claim, clinicians also agreed that the most feasible time to collect additional biopsies for organoid research would be at surgical resection (after neoadjuvant chemotherapy), although this preference for surgical intervention may have been influenced by the high proportion of surgical specialists surveyed in comparison to gastroenterologists or medical oncologists that are more heavily involved with endoscopic tissue acquisition and treatment selection, respectively. Furthermore, the ideal scenario based on these clinicians’ perceptions would be to collect biopsies at surgical resection from patients who underwent neoadjuvant therapy and then personalize adjuvant treatment based on organoid drug sensitivities. This tissue collection time point is currently the most common across published studies (Li et al., 2022; Seidlitz et al., 2019; Steele et al., 2019; Xu et al., 2024; Yan et al., 2018; Zhang et al., 2023; Zu et al., 2023). However, only a limited number of existing studies include the assessment of organoid drug responses with patients’ clinical response to the same drug(s), with only two studies examining adjuvant treatment in fewer than 10 patient-matched organoids (Ahn et al., 2014; Zu et al., 2023). Clearly, larger cohorts are needed to strengthen this area of research, with the implication from these survey results that the development of organoid-guided treatment for GC should focus on surgical resection for tissue collection and testing to inform adjuvant treatment choice.

The time required to produce organoid drug test results is a major aspect in determining if organoids could be feasibly integrated into standard clinical care. Very few current GC organoid studies have reported the time required to provide organoid drug results, and of those that do, the time varies from 2 to 3 weeks (Schmäche et al., 2024; Zhao et al., 2024). Only Schmäche et al. clearly described a 3-week time frame, which is from biopsy receipt to the first drug test result (Schmäche et al., 2024). To guide adjuvant treatment decisions, 44% of the clinicians indicated they would need results within 4–6 weeks of surgical resection, followed by 31% recording 2–4 weeks. This timeline fits with those of other studies examining optimal adjuvant therapy for GC, with 55.6% (*n* = 467) of patients receiving adjuvant chemotherapy after 4–8 weeks (Park et al., 2015), and treatment initiated within 6 weeks significantly improving 5-year survival compared to later initiation (Kim et al., 2025). This timing is consistent with the turnaround time reported by the two largest organoid studies in the GC field to date (Schmäche et al., 2024; Zhao et al., 2024). However, in real-world settings, this might be hindered by other factors such as low organoid culture success rates of approximately 67% that can be dependent on tissue quality, quantity, and prior treatment exposure, indicating that about one-third of patients would not benefit from this technology (Silva et al., 2024). Reproducibility is another major challenge with a lack of standardized protocols for testing and interlaboratory variability. Cost and infrastructure also remain significant challenges to incorporate organoid technology into clinical settings, as organoids require relatively advanced technical expertise and expensive materials, including 3D matrix, growth factors, dedicated cell culture spaces, and biobanking capabilities. However, research is underway to address this, with studies establishing miniaturized organoid methods to introduce high-throughput approaches with automation and short turnaround times, requiring less starting material (Du et al., 2020; Hirt et al., 2022). However, these advances remain to be tested for efficacy and health economics advantages in larger cohorts in real-world settings. Overall, these survey responses, along with previous studies, highlight that clinicians estimate they would need to receive organoid-informed drug results within 4–6 weeks of surgery to guide adjuvant treatment decisions for patients with GC. Researchers working in the organoid field should use this time frame as a guide in future studies.

Clinicians also indicated that organoids could be helpful for patients with metastatic GC to guide treatment decision-making when patients fail first-line systemic treatment (i.e., in a refractory palliative setting). There is evidence that organoids can be established from metastatic tissue or ascitic fluid in other cancers, including colorectal, pancreatic, prostate, and breast cancers (Buzzelli et al., 2018; Gao et al., 2014; Narasimhan et al., 2020; Sachs et al., 2018; Tiriac et al., 2018; Vlachogiannis et al., 2018; Weeber et al., 2015). A few studies in GC have established organoids from malignant ascites or metastatic lymph nodes (Li et al., 2019; Yan et al., 2018), but there is a need for more comprehensive research in establishing organoids from metastatic sites in GC. Understandably, obtaining repeat biopsies at the time of progression is not always possible and was predominantly recorded as “somewhat feasible” by clinicians in our survey, suggesting this may not always be a feasible cohort for the use of organoid-guided personalized medicine.

Analysis of the qualitative comments section revealed six themes, and most of these comments were research-related concerns, suggestions, or standalone comments. A key concern for clinicians was organoid culture success rates that can limit personalized treatment access for some patients. This is a valid concern, and a key objective of current GC organoid studies is to increase culture success rates, with researchers noting contamination issues and normal organoid overgrowth as problems to be addressed (Silva et al., 2024). Another concern was the lack of immune cells in current GC organoid cultures that limits the personalization of immunotherapies for patients. While current organoid studies have focused on correlating chemotherapeutic and targeted therapeutic responses between organoids and patients, research is underway to co-culture immune, vascular, and fibroblast cells with epithelial cells to improve organoid systems (Wang et al., 2025). Thus, these survey data reinforce current research efforts to develop complete organoid systems that replicate most parts of the patients’ original tumor as a key major focus.

While research is improving, clinicians highlighted the importance of standardizing organoid drug testing, emphasizing that these assays should be officially validated/certified and that guidelines should be in place to reduce variability between results. Standardizing organoid modeling has been discussed in great detail in the past (Lee et al., 2024; Zhou et al., 2023), exemplified by the recent efforts made by the US National Institutes of Health (NIH) in launching the Standardized Organoid Modeling Center (SOM), which aims to develop standardized organoid-based “new approach methodologies” by using advanced technologies such as artificial intelligence. The cost of organoid drug testing was another concern for clinicians, thus emphasizing the need to address this if organoids are to be integrated into standard care. To our knowledge, none of the current GC organoid studies have mentioned the cost related to organoid drug assays. Promising cost reduction strategies have been observed in pancreatic organoid research (Du et al., 2020; Hirt et al., 2022) with the use of fully automated robotic systems to conduct drug assays, which could help reduce the variability, number of organoids, and volume of reagents required for drug assays, leading to decreased costs. Reporting this in future organoid studies could be a guide to better understand the factors related to cost. Overall, clinicians highlighted organoid culture success rates, co-culture models, standardized organoid practices, and cost as research-related factors to be addressed in future organoid platform development.

To be applied to clinical settings, many clinicians noted the importance of large clinical trials to demonstrate the effectiveness of organoid-guided treatment selection, with one emphasizing the importance of demonstrating superior patient outcomes beyond organoid-to-patient treatment correlations. While many clinical trials have been completed in other cancers (lung, pancreatic, breast, and colorectal) correlating patient treatment responses (NCT04859166, NCT05351983, NCT04450706, NCT05384184), the majority of clinical trials are actively recruiting patients. Furthermore, in the field of GC, there are registered clinical trials that are currently recruiting patients to predict treatment responses (NCT05652348, NCT06100003), with one completed clinical trial registered as NCT03429816. This completed study “Outcome prediction of systemic treatment in esophagogastric carcinoma” (Opposite) successfully matched organoid response with clinical responses for 12 patients, demonstrating that organoids may have the ability to personalize treatment in GC patients (Schmäche et al., 2024). However, the field still requires larger clinical trials that personalize treatment in patients based on the organoid drug test results. Clinicians also highlighted the importance of increased access to organoid data and education, which could be addressed in future research.

In addition, clinicians emphasized the importance of political support and ethics related to organoid research. While cerebral organoids are in the spotlight due to concerns about conscience, sentience, and moral status from patients’ perspectives, there’s an interest in ethics related to all organoids in general (Ravn et al., 2023). Therefore, moving forward with GC organoid research, we need to consider appropriate governance by including certified organoid drug testing processes, medicolegal validity, political/institutional support, and ethical oversight to ensure responsible use of organoids.

The limitations of this survey include selection bias due to disproportionate responses from Australia and Europe compared with other continents of the world. The incidence and prevalence of GC-related mortality are highest in East Asia; however, we received only one response from Japan, limiting input from a key cohort of clinicians. East Asian GC guidelines prioritize early curative, extensive surgery, with systemic therapy more often administered in the post-operative setting. Thus, organoid technology is unlikely to provide clinical utility in East Asia in the neoadjuvant setting. However, the timing of adjuvant therapy after surgery is broadly similar across the globe; hence, had more clinicians from East Asia been included in the survey, they may have perceived value in use of organoid technology to personalize treatment in the adjuvant setting (Stroobant et al., 2025). Furthermore, we received one response from North America, thus not sufficiently covering this region of the world. The sample size is relatively small; hence, more research is required to be able to extend these results to a broader group of clinicians. In addition, clinicians with prior knowledge of organoids may have been more likely to participate in the survey and express positive attitudes, potentially introducing response bias. As such, how accurately results represent the broader GC clinical community is uncertain. These clinician perspectives related to time sensitivity or patient selection for organoid assays may not be translatable to other cancer types, as this is a GC-specific survey; however, the suggestions and concerns revealed via the six themes of the qualitative comments may apply to other cancer organoid fields.

In conclusion, this international survey explored clinicians’ perspectives on the role of GC organoids in precision medicine. Given the clinicians’ substantial awareness of organoid research, their responses suggested that they perceived GC organoids as being most useful to personalize treatment in the adjuvant setting for patients with poor response to first-line treatment in the future. They favored the need for organoid drug test results within 4–6 weeks of surgical resection, providing an important benchmark for researchers in the field of organoid platform development. They also emphasized the need for certified organoid testing, clinical trials, and the policies/ethics surrounding the use of organoids, while raising concerns and offering suggestions regarding current research challenges within the organoid field that can be used to guide organoid platform development. Overall, this clinician survey highlights important aspects of organoid-related research, noting the best patient cohort that would benefit from organoid-guided treatment, time sensitivity, and other challenges and benefits of using organoids to guide personalized treatment in GC patients.

## Resource availability

### Lead contact

Requests for further information should be directed to and will be fulfilled by the corresponding author, Susan L. Woods (susan.woods@adelaide.edu.au).

### Materials availability

The datasets supporting the conclusions of this article are included within the article and its supplemental information. For any further requests, please contact the corresponding author.

### Data and code availability

All data reported in this paper will be shared by the lead contact upon request. This paper does not report original code. Any additional information required to reanalyze the data reported in this paper is available from the lead contact upon request.

## Consortia

Members of the Gastric Cancer Organoid Survey group (GCOSG) are David S. Liu, Ignace De Hingh, Neda Nikolic, Megan Barnet, Manjunath Siddaiah-Subramanya, David I. Watson, Marcello Guaglio, Diane Goéré, Chirag Patel, Conrad Stranz, Markus Trochsler, Philip Game, Tim Bright, Lauren Kennedy, Li L. Kuan, Guy Maddern, Shalvin Prasad, Matthew Watson, Cameron Schauer, Harleen Kaur, Michael W. Hii, Marina Puchinskaya, Radu Vidra, William Butterworth, Karez Namiq, Jane M. Andrews, Tudor Boloș, Emiliana-Viorica Larionesi, Matthew A. R. Stokes, Daniel Worthley, Jessie A. Elliott, Andreas R. R. Weiss, Yit J. Leang, Benjamin Tan, Munir Tarazi, Hien Le, Stephen Kunz, Di Maggio Francesco, Charles J. Rayner, Martin Harutyunyan, Patricia Kaazan, Richard Heddle, Nagendra N. Dudi-Venkata, Vijay Abraham, Julian Süsstrunk, Aniket Nadkarni, Panagiotis J. Vlachostergiostergios, Shantanu Joglekar, Oktay Irkorucu, Brian Stein, Semra D. Atici, Vladimir Andelkovic, Mariella Spalato-Ceruso, Sharif Ahmed, Ryan Newbold, Michele Ghidini, Motohiro Imano, Birute Brasiuniene, İmdat Eroğlu, Fabio M. Oddi, Christopher M. Jones, Martynas Luksta, Samuel Smith, Sharon Pattison, Shailesh V. Shrikhande, Tharani Krishnan, Savio G. Barreto, Manju D. Chandrasegaram, Bartłomiej Tomasik, Andrew L. Nathanson, Piotr T. Wysocki and Sam Banks.

## Acknowledgments

We would like to sincerely thank the GCOSG for their expertise that was important to the successful completion of this survey project. We also thank the 10.13039/100004437EACR, GI Cancer Trials, 10.13039/501100001202GESA, NZSG, AANZGOSA, PSOGI, and 10.13039/501100007075ESMO for their support in endorsing the survey and facilitating the distribution of the survey to their members. This work was supported by the Gastroenterological Society of Australia Bushell Post-Doctoral Research Fellowship (SLW), the College of Health at the Adelaide University (SLW). T.N.S. is supported by the Adelaide University Research Scholarship.

## Author contributions

T.N.S. contributed to the manuscript conception and writing of the manuscript; S.L.W., J.A.W., N.J.C., D.S.L., H.K., and T.K. reviewed and edited the manuscript before submission and approved the final manuscript. All authors have read and agreed to the published version of the manuscript.

## Declaration of interests

The authors declare no competing interests.

## STAR★Methods

### Key resources table

**Table:** 

REAGENT or RESOURCE	SOURCE	IDENTIFIER
**Software and algorithms**		

Survey managed using electronic database	REDcap hosted at Adelaide University	N.A.
Data analysis	Microsoft Excel (Microsoft Corporation, Redmond, WA, USA)	N.A.
Data Analysis	GraphPad Prism (version 10.0.0 for Windows, GraphPad Software, Boston, Massachusetts, USA)	N.A.

### Experimental model and study participant details

The ethics for this survey have been reviewed and approved by the Central Adelaide Local Health Network (CALHN) Human Research Ethics Committee (CALHN HREC: 21006). The study was conducted in accordance with the National Health and Medical Research Council (NHMRC) National Statement on Ethical Conduct in Human Research (2023). Implied consent of participants was acquired through confirmation that they had read and understood the information in the survey participant information sheet and agreed to participate voluntarily.

### Method details

The survey was managed using the REDCap electronic database hosted at Adelaide University. The survey was composed of five sections, with a total of 14 questions, multiple choice, matrix format and short answer questions. Questions were focused on general information about the clinician (country of practice and specific profession), their knowledge of organoid technology, their views on optimal patient selection for future organoid-guided treatment, time sensitivity, and future potential feasibility of organoids in clinical practice. Distributed survey is included in supplemental information.

The survey was distributed to local and national Australian clinicians via institutional and hospital based lists, globally through membership of clinical societies including European Association for Cancer Research (EACR), GI Cancer Trials (formerly Australasian gastrointestinal Trials Group), Gastroenterological society of Australia (GESA), New Zealand Society of Gastroenterology (NZSG), Australia & Aotearoa New Zealand Gastric & Esophageal Surgery Association (AANZGOSA), The Peritoneal Surface Oncology Group International (PSOGI), European Society for Medical Oncology (ESMO) and directly using LinkedIn to identify international UGI clinicians.

### Quantification and statistical analysis

The survey was opened from May to December 2025, once closed data were analyzed using Microsoft Excel (Microsoft Corporation, Redmond, WA, USA) and GraphPad Prism (version 10.0.0 for Windows, GraphPad Software, Boston, Massachusetts, USA). The response rate could not be calculated as the survey was disseminated to large national and international societies where the total number of recipients could not be tracked. A simplified version of Braun and Clarke’s thematic analysis framework was used to analyze the qualitative comments (Braun and Clarke, 2020). Briefly, the qualitative survey comments were read multiple times to achieve familiarity and to identify recurring ideas, patterns, and similarities across participant responses. Sections of text expressing similar meanings were then assigned a code prior to grouping these into six themes including research, clinical, cost, access, policy, and ethical by one investigator. As independent coding by a second reviewer was not performed, inter-rater reliability could not be assessed, representing a potential limitation of the qualitative analysis.

## Supplemental information

Document S1. Figures S1 and S2

Document S2. Article plus supplemental information

## Footnotes

Supplemental information can be found online at https://doi.org/10.1016/j.stemcr.2026.103006.
