# Supplementary material for: The role of gastric cancer patient-derived organoids in precision medicine: Clinicians’ perspective
Source: Stem Cell Reports. 2026 Jul 9;21(8):103006. doi: 10.1016/j.stemcr.2026.103006 (PMC13476899; doi:10.1016/j.stemcr.2026.103006)
Supplement: Supplementary file 1 [file mmc1.pdf]

Stem Cell Reports, Volume *21*

Supplemental Information

The role of gastric cancer patient-derived organoids in precision medicine: Clinicians' perspective

Tharindie N. Silva, Harleen Kaur, Tharani Krishnan, David S. Liu, Margaret Lee, Markus Trochsler, Nicholas J. Clemons, Josephine A. Wright, Susan L. Woods, and Gastric Cancer Organoid Survey group

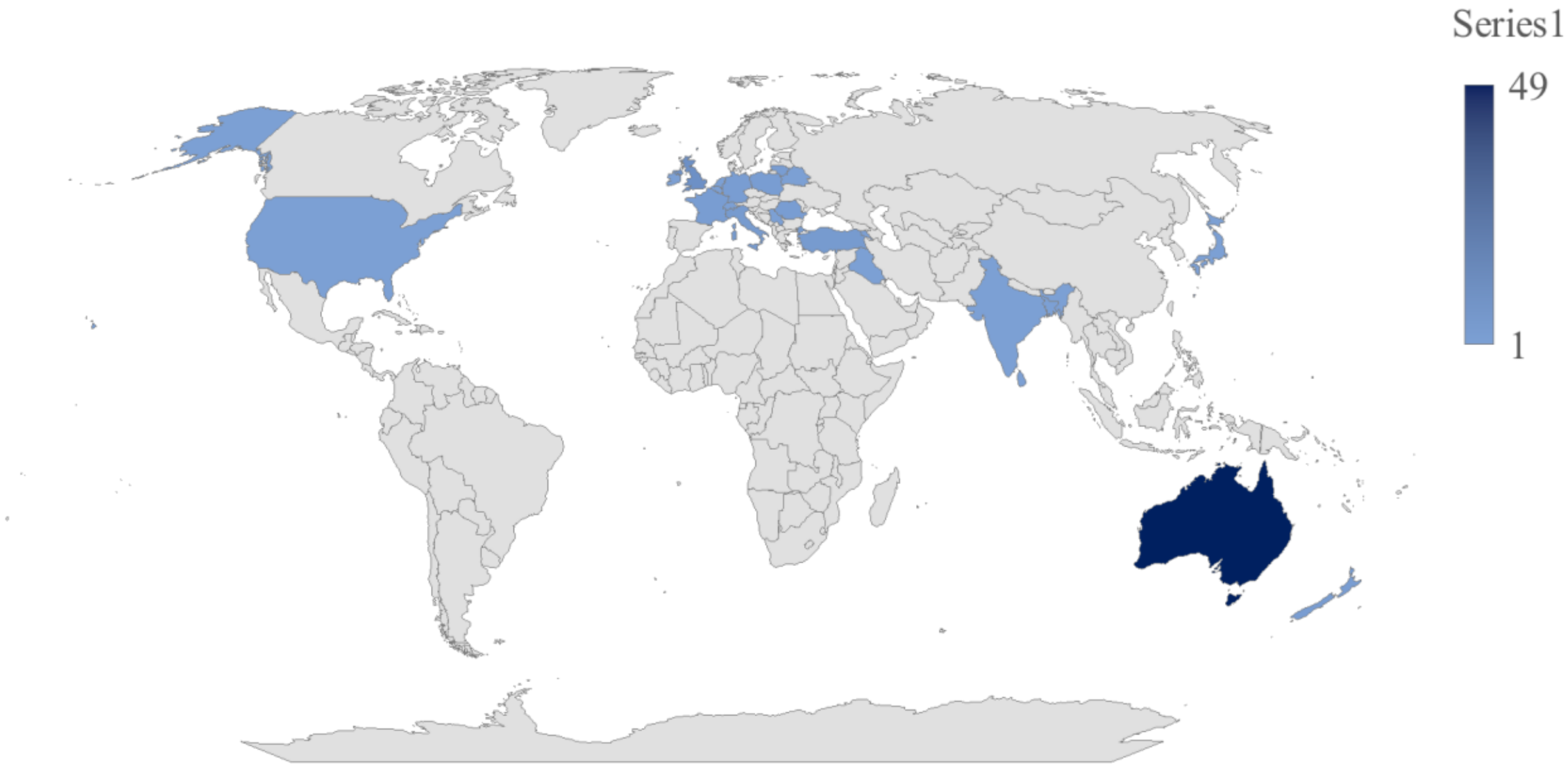

Figure S1. Geographic distribution of survey respondents, representing 23 countries across four continents.

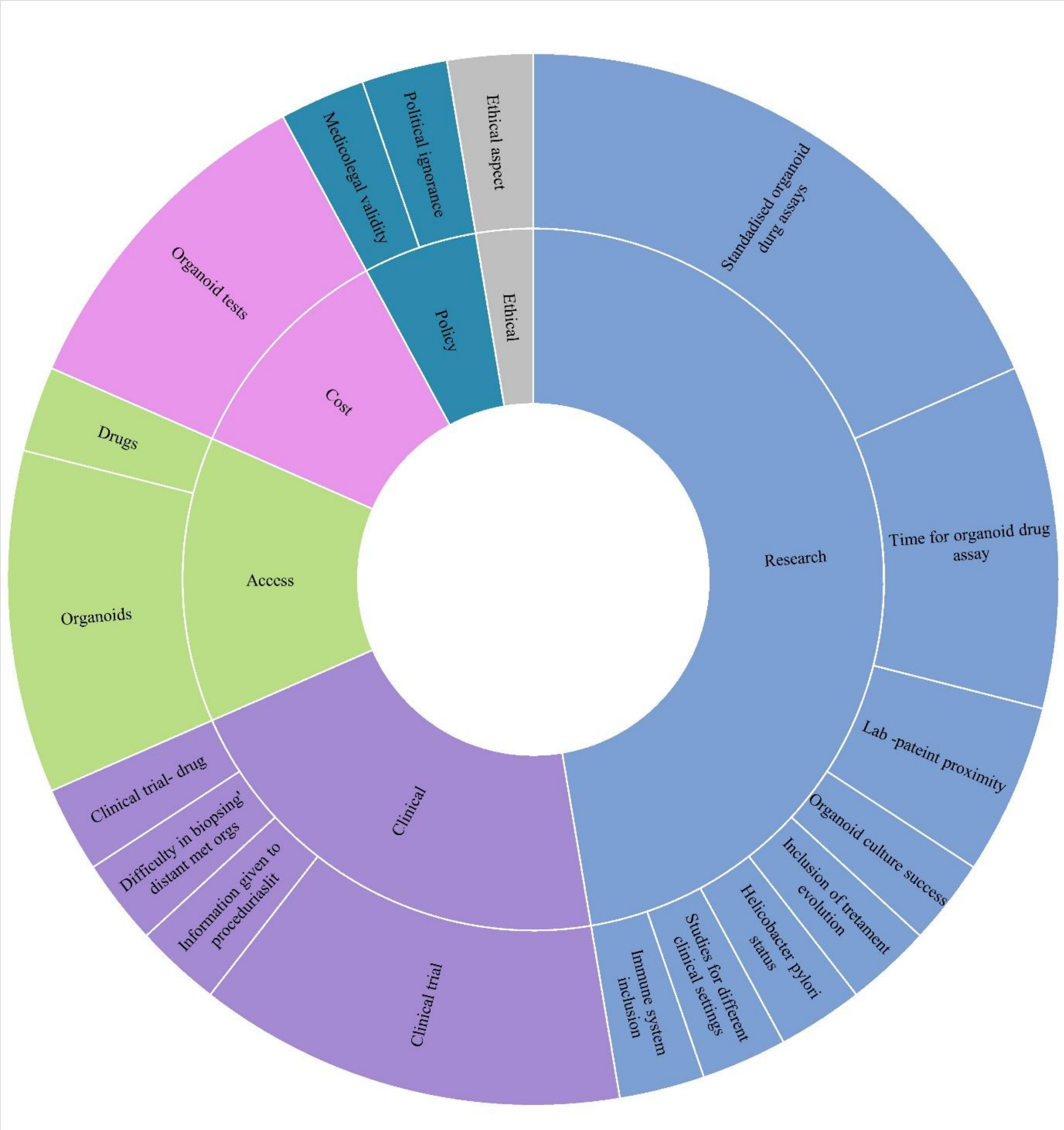

Figure S2. Overview of the six key themes identified through qualitative analysis of clinician responses.

The role of gastric cancer patient-derived organoids in precision medicine: A Clinician's perspective.

Purpose of this survey

The goal of this survey is to capture clinicians' insights on the potential of organoid models in guiding treatment for non-curative gastric cancer patients.

Survey completion and authorship

It will take approximately 5- 7 minutes to complete the survey.

At the end of the survey, you will have the option to provide your name and email address. By completing the survey and sharing your details, your participation will be acknowledged as part of the Gastric Cancer Organoid Survey group, with authorship on any resulting publications.

Personal details are not linked to survey responses, and all responses remain anonymous.

Participant information sheet

Here's the link to the Participant Information Sheet for further details PIS.pdf

By continuing with the survey, you provide your implied consent, confirming that you have read and understood the information and agree to participate voluntarily under the outlined conditions.

Click the "Next" button to proceed to the survey.

Thank you for your interest in participating in this study.

The role of gastric cancer patient-derived organoids in precision medicine: A Clinician's perspective.

Section 1: General Information

1 In which country do you practice?

◯ Afghanistan
◯ Albania
◯ Algeria
◯ Andorra
◯ Angola
◯ Antigua and Barbuda
◯ Argentina
◯ Armenia
◯ Australia
◯ Austria
◯ Azerbaijan
◯ Bahamas
◯ Bahrain
◯ Bangladesh
◯ Barbados
◯ Belarus
◯ Belgium
◯ Belize
◯ Benin
◯ Bhutan
◯ Bolivia
◯ Bosnia and Herzegovina
◯ Botswana
◯ Brazil
◯ Brunei
◯ Bulgaria
◯ Burkina Faso
◯ Burundi
◯ Cabo Verde
◯ Cambodia
◯ Cameroon
◯ Canada
◯ Central African Republic
◯ Chad
◯ Chile
◯ China
◯ Colombia
◯ Comoros
◯ Congo (Congo-Brazzaville)
◯ Costa Rica
◯ Croatia
◯ Cuba
◯ Cyprus
◯ Czech Republic (Czechia)
◯ Denmark
◯ Djibouti
◯ Dominica
◯ Dominican Republic
◯ Ecuador
◯ Egypt
◯ El Salvador
◯ Equatorial Guinea
◯ Eritrea
◯ Estonia
◯ Eswatini
◯ Ethiopia
◯ Fiji
◯ Finland
◯ France
◯ Gabon
◯ Gambia
◯ Georgia
◯ Germany
◯ Ghana
◯ Greece
◯ Grenada
◯ Guatemala
◯ Guinea
◯ Guinea-Bissau

◯ Guyana
◯ Haiti
◯ Honduras
◯ Hungary
◯ Iceland
◯ India
◯ Indonesia
◯ Iran
◯ Iraq
◯ Ireland
◯ Israel
◯ Italy
◯ Jamaica
◯ Japan
◯ Jordan
◯ Kazakhstan
◯ Kenya
◯ Kiribati
◯ Korea, North
◯ Korea, South
◯ Kuwait
◯ Kyrgyzstan
◯ Laos
◯ Latvia
◯ Lebanon
◯ Lesotho
◯ Liberia
◯ Libya
◯ Liechtenstein
◯ Lithuania
◯ Luxembourg
◯ Madagascar
◯ Malawi
◯ Malaysia
◯ Maldives
◯ Mali
◯ Malta
◯ Marshall Islands
◯ Mauritania
◯ Mauritius
◯ Mexico
◯ Micronesia
◯ Moldova
◯ Monaco
◯ Mongolia
◯ Montenegro
◯ Morocco
◯ Mozambique
◯ Myanmar (Burma)
◯ Namibia
◯ Nauru
◯ Nepal
◯ Netherlands
◯ New Zealand
◯ Nicaragua
◯ Niger
◯ Nigeria
◯ North Macedonia
◯ Norway
◯ Oman
◯ Pakistan
◯ Palau
◯ Panama
◯ Papua New Guinea
◯ Paraguay
◯ Peru
◯ Philippines
◯ Poland
◯ Portugal
◯ Qatar
◯ Romania

◯ Russia
◯ Rwanda
◯ Saint Kitts and Nevis
◯ Saint Lucia
◯ Saint Vincent and the Grenadines
◯ Samoa
◯ San Marino
◯ Sao Tome and Principe
◯ Saudi Arabia
◯ Senegal
◯ Serbia
◯ Seychelles
◯ Sierra Leone
◯ Singapore
◯ Slovakia
◯ Slovenia
◯ Solomon Islands
◯ Somalia
◯ South Africa
◯ South Sudan
◯ Spain
◯ Sri Lanka
◯ Sudan
◯ Suriname
◯ Sweden
◯ Switzerland
◯ Syria
◯ Taiwan
◯ Tajikistan
◯ Tanzania
◯ Thailand
◯ Timor-Leste
◯ Togo
◯ Tonga
◯ Trinidad and Tobago
◯ Tunisia
◯ Turkey
◯ Turkmenistan
◯ Tuvalu
◯ Uganda
◯ Ukraine
◯ United Arab Emirates
◯ United Kingdom
◯ United States
◯ Uruguay
◯ Uzbekistan
◯ Vanuatu
◯ Vatican City
◯ Venezuela
◯ Vietnam
◯ Yemen
◯ Zambia
◯ Zimbabwe

2	Which of the following best describes your professional role?	◯ Medical oncologist<br>◯ Medical oncologist trainee<br>◯ Surgeon<br>◯ Surgical Trainee<br>◯ Gastroenterologist<br>◯ Gastroenterology Trainee<br>◯ Radiation oncologist<br>◯ Radiation Oncology Trainee<br>◯ Other (Please specify) ______

Section 2: Current Use of Organoids in Clinical Practice

Organoid culture is a method used to grow "mini organs in a dish" derived from stem cells. Tumour organoids are derived from cancer cells and represent personalised models that can be utilised for research and drug testing

3 Are you aware of any research that demonstrates the use of organoids in predicting treatment response for cancer patients?

- ◯ Yes
- ◯ Maybe
- ◯ No

4 Do you believe organoids could help predict how a patient's cancer will respond to specific drugs?

- ◯ Strongly agree
- ◯ Agree
- ◯ Neutral
- ◯ Disagree
- ◯ Strongly disagree
- ◯ Unsure

Section 4: Patient Selection for Organoid Use

Patient-derived gastric cancer organoids can be used in vitro with anti-cancer drugs to help personalise and guide treatment decisions.

5 Please rate the feasibility of taking additional biopsies for organoid cultures.

	Feasible	Somewhat feasible	Not feasible
At diagnosis	◯	◯	◯
At restaging endoscopy (post neoadjuvant therapy)	◯	◯	◯
At surgical resection	◯	◯	◯
Loco regional recurrence	◯	◯	◯
At diagnosis of de novo metastatic disease in liver	◯	◯	◯
At diagnosis of de novo metastatic disease in peritoneum	◯	◯	◯
At diagnosis of de novo metastatic disease in lungs	◯	◯	◯
Palliative setting first line	◯	◯	◯
Palliative setting second line and beyond	◯	◯	◯

6 Please rank the following 4 clinical scenarios based on where organoid models could provide the most value in guiding treatment options

	Most valuable			Least valuable
After poor response to front line neoadjuvant treatment, to guide second line treatment of recurrent disease	◯	◯	◯	◯
For palliative treatment in metastatic disease	◯	◯	◯	◯
For front line treatment in patients who present with metastatic disease.	◯	◯	◯	◯
For targeted treatment in HER2 positive disease	◯	◯	◯	◯

7 Would you consider using organoid drug testing to guide treatment choice for patients who fail first-line standard-care treatment?

◯ Yes
◯ No
◯ Unsure

8 Would you consider using organoid drug testing to guide treatment decisions for patients in the refractory palliative setting after failing standard of care treatments?

◯ Yes
◯ No
◯ Unsure

Section 5: Time Sensitivity and Need for Results

9 If organoids were used to guide treatment decisions in the adjuvant setting, what is the maximum time after surgical resection that you would consider acceptable for receiving organoid-informed drug results?

- ◯ Within 1-2 weeks
- ◯ Within 2-4 weeks
- ◯ Within 4-6 weeks
- ◯ Within 6-8 weeks
- ◯ Within 8-12 weeks
- ◯ Other (please specify) ______

10 What is the maximum time from diagnosis to the availability of organoid-informed drug testing results that would be useful to guide the choice of treatment for a patient with advanced gastric cancer?

- ◯ Within 1-2 weeks
- ◯ Within 2-4 weeks
- ◯ Within 4-6 weeks
- ◯ Within 6-8 weeks
- ◯ Within 8-12 weeks
- ◯ Other (please specify) ______

11 If organoids could help identify the most effective chemotherapy for a patient, how would you rank the importance of the following factors before starting treatment with an organoid-test-guided drug?

	Most important					Least important
Cost of drug(s)	◯	◯	◯	◯	◯	◯
Availability of drug(s)	◯	◯	◯	◯	◯	◯
Safety/tolerability profile of drug(s)	◯	◯	◯	◯	◯	◯
Prior experience using the drug(s)	◯	◯	◯	◯	◯	◯
Health status of patient	◯	◯	◯	◯	◯	◯
Availability of a clinical trial	◯	◯	◯	◯	◯	◯

Do you have any other comments or factors that you think should be considered before using an organoid-based treatment test? ________________________________

Section 6: Future Potential and Feasibility of Organoids in Clinical Practice

12 Please rate the importance of factors below for incorporating organoid test results as a standard part of your gastric cancer treatment decision-making.

	Very Important	Important	Not Important
Demonstrated clinical effectiveness of organoid test	◯	◯	◯
Palliative treatment only	◯	◯	◯
Increased access to organoid research data and education	◯	◯	◯
Clinical laboratories certified organoid testing processes	◯	◯	◯
Organoid drug test cost	◯	◯	◯

Are there any other important factors to consider when incorporating organoid test results into standard gastric cancer treatment? __________________________________

13 In your opinion, how likely is it that organoids will become a standard tool for personalising treatment in gastric cancer within the next 10 years?

Very Likely — Neutral — Very Unlikely

(Place a mark on the scale above)

14 Do you have any other comments or suggestions regarding the use of organoids in gastric cancer treatment prediction? __________________________________

You've completed all the questions. Click submit to proceed to the final page.

The role of gastric cancer patient-derived organoids in precision medicine: A Clinician's perspective.

Contact Information for Authorship Acknowledgment

Would you like your participation to be acknowledged as part of the Gastric Cancer Organoid Survey group, with authorship on any resulting publications?

◯ Yes
◯ No

Your participation in this study is greatly appreciated.

Thank you!

Please provide the name you wish to have listed in the published paper. ______________________

Please provide your email address for further communication. ______________________

Thank you for completing the survey.

You can now submit this form.

Note: If you return to the survey and make changes to your contact information, only your most recent entry will be recorded.
